# Is Wearable Technology Becoming Part of Us? Developing and Validating a Measurement Scale for Wearable Technology Embodiment

**DOI:** 10.2196/12771

**Published:** 2019-08-09

**Authors:** Elizabeth C Nelson, Tibert Verhagen, Miriam Vollenbroek-Hutten, Matthijs L Noordzij

**Affiliations:** 1 Biomedical Signals and Systems University of Twente Enschede Netherlands; 2 Center for Market Insights Amsterdam University of Applied Sciences Amsterdam Netherlands; 3 Ziekenhuis Groep Twente Almelo Netherlands; 4 Department of Psychology, Health and Technology University of Twente Enschede Netherlands

**Keywords:** embodiment, wearable technology, measurement development, human technology interaction, eHealth, mHealth, wearable electronic devices, self-help devices, health information technology, medical informatics

## Abstract

**Background:**

To experience external objects in such a way that they are perceived as an integral part of one’s own body is called embodiment. Wearable technology is a category of objects, which, due to its intrinsic properties (eg, close to the body, inviting frequent interaction, and access to personal information), is likely to be embodied. This phenomenon, which is referred to in this paper as *wearable technology embodiment*, has led to extensive conceptual considerations in various research fields. These considerations and further possibilities with regard to quantifying *wearable technology embodiment* are of particular value to the mobile health (mHealth) field. For example, the ability to predict the effectiveness of mHealth interventions and knowing the extent to which people embody the technology might be crucial for improving mHealth adherence. To facilitate examining *wearable technology embodiment*, we developed a measurement scale for this construct.

**Objective:**

This study aimed to conceptualize wearable technology embodiment, create an instrument to measure it, and test the predictive validity of the scale using well-known constructs related to technology adoption. The introduced instrument has 3 dimensions and includes 9 measurement items. The items are distributed evenly between the 3 dimensions, which include body extension, cognitive extension, and self-extension.

**Methods:**

Data were collected through a vignette-based survey (n=182). Each respondent was given 3 different vignettes, describing a hypothetical situation using a different type of wearable technology (a smart phone, a smart wristband, or a smart watch) with the purpose of tracking daily activities. Scale dimensions and item reliability were tested for their validity and Goodness of Fit Index (GFI).

**Results:**

Convergent validity of the 3 dimensions and their reliability were established as confirmatory factor analysis factor loadings (>0.70), average variance extracted values (>0.50), and minimum item to total correlations (>0.40) exceeded established threshold values. The reliability of the dimensions was also confirmed as Cronbach alpha and composite reliability exceeded 0.70. GFI testing confirmed that the 3 dimensions function as intercorrelated first-order factors. Predictive validity testing showed that these dimensions significantly add to multiple constructs associated with predicting the adoption of new technologies (ie, trust, perceived usefulness, involvement, attitude, and continuous intention).

**Conclusions:**

The wearable technology embodiment measurement instrument has shown promise as a tool to measure the extension of an individual’s body, cognition, and self, as well as predict certain aspects of technology adoption. This 3-dimensional instrument can be applied to mixed method research and used by wearable technology developers to improve future versions through such things as fit, improved accuracy of biofeedback data, and customizable features or fashion to connect to the users’ personal identity. Further research is recommended to apply this measurement instrument to multiple scenarios and technologies, and more diverse user groups.

## Introduction

There has been an impressive increase in the usage of wearable technologies, digital devices that incorporate wireless connectivity and allow the user to seamlessly access, interact with, and exchange information anywhere and anytime [1], since their introduction into the marketplace [2,3]. Devices such as smart phones, activity trackers, and smart watches have been widely embraced [2,3] and seem to have become almost inseparable from the human body. Although various research fields address this phenomenon from their own perspectives, there seems to be consensus that users can interact with technology, accepting it as part of them and even experience it as part of their body [4]. Beyond body extension, users can extend their cognitive performance [5,6] through the constant access to information [7], self-identity [8-12], and through the highly personal experience [13,14] tailoring it to their personal preferences [15]. However, studies addressing this form of embodiment represent a relatively new area of focus and more empirical research is called for [4,16,17]. In addition, no study has yet combined the different embodiment experiences (body, cognition, and sense of self) to cover the full spectrum of the individual.

The embodiment experiences could be highly relevant for the study and use of wearable technology in health care. A substantial body of existing research addressed wearable technology in, among others, studies into health information recording [18,19], mood, sleep [20], personal sensing and biofeedback in mental health care [21-23], remote patient monitoring [24], medication adherence [25], and technology-assisted procedures [26]. Furthermore, there is an increasing use of wearable technologies that seamlessly fit into the user’s everyday lifestyle, can be worn on the body or mated with human skin, and continuously and closely monitor the user’s motion and vital signs (eg, pulse and blood pressure) [20,27], and as such provides the user with the information needed for self-assessment and change in health behaviors and health outcomes [18,28,29]. Generating insights into embodiment experiences and how to measure it may prove crucial for researchers and practitioners to further their understanding of what drives users to keep wearing the technology on the long term and be adherent to the health coaching associated with its measurements.

Past research on devices such as virtual reality [16] or cognitive prostheses [30] have addressed the embodiment of technology but were unable to measure it because of the lack of a measurement instrument [4,16,17]. In this paper, we aim to address this gap in the literature by proposing and validating the concept of *wearable technology embodiment* and operationalize it by developing a valid measurement instrument utilizing established conceptualization and measurement procedures [31]. This delineation implies that researchers and practitioners can use the instrument to measure the embodiment of some of the most widely adopted wearable technologies in the market today such as smartphones, activity trackers, and smart watches, as well technologies such as smart clothing/jewelry, head-mounted displays, and ear-worn technology [32].

## Methods

### Scale Development Procedure

To develop and test a measurement instrument for wearable technology embodiment, we followed an established scale development procedure [31,33-36] (Table 1). Scale development is a recognized process for developing and validating a definition and measurement scale for a construct that cannot be adapted from a similar scale or does not yet exist.

**Table 1:** Overview of scale development procedure.

Step and description		Actions undertaken in this study
1	Conceptualization: develop a conceptual definition of the construct	Conceptualization of target construct; scoping review; study selection; data extraction; define property; define entity; establish dimensionality of construct; construct definition
2	Development of measures: generate items to represent the construct and assess the content validity of the items	Item generation and sorting; expert interviews; item refinement
3	Method of validation: formally specify the measurement model	Formally specify the measurement model; include dependent variables for measurement
4	Scale evaluation and refinement: collect data, scale purification and refinement	Evaluate goodness of fit; assess validity at the construct level; assess reliability at the item level; eliminate problematic indicators
5	Validation: assess scale validity	Assess convergent validity; assess discriminant validity; test alternative models; test predictive validity

### Step 1: Conceptualization

#### Conceptualization of Target Construct, Literature Review and Study Selection

To begin, we conducted a scoping review focused on the specific experience of embodiment of technologies worn or carried, for example, mobile phones or smart watches, searching for terms describing the experience of embodiment with a tool (Textbox 1). The scoping review included: (1) identifying the research question; (2) identifying relevant studies; (3) study selection; (4) charting the data; (5) collating, summarizing, and reporting the results; and (6) consultation [37,38]. Our review resulted in a total of 80 papers in disciplines that included electronic health, neuroscience, mobile computing, wearable computing, ubiquitous computing, psychology, sociology, and philosophy. Although some areas specifically describe the embodiment of technology, other research described the embodiment of tools such as Merleau Ponty’s example of a blind person embodying their walking cane [39]. We believe these examples also apply to the embodiment of technologies. Therefore, studies which included the embodiment of a technical or nontechnical tool were included in the review. In total, 20 of the papers were discarded because they described embodiment without the use of technology or tools and therefore did not help in developing the concept. The 60 remaining papers were organized and analyzed for their description of the embodiment of technology/tools.

**Search terms used in scoping review.**

Google Scholar, Science Direct, Science.gov, SpingerLink, WorldWideScience, JSTOR, and Web of Science

- Embodiment
  - AND (tool OR technology OR digital OR wearable OR mobile OR cognitive)
- Embodied interaction
  - AND (tool OR technology OR digital OR wearable OR mobile)
- Prosthesis
  - AND (cognitive OR embodiment OR technology OR digital OR wearable OR mobile)
- Phenomenology
  - AND **(**wearable OR mobile OR digital OR technology tool OR cognitive)

#### Data Extraction and Establishing the Entity and the Property

A structure was utilized to organize the study characteristics of the 60 papers, including: (1) authors and publication year, (2) main research findings, (3) research design details *(ie, experimental or nonexperimental design)*, (4) item embodied (ie, mobile and wearable technology or physical prosthetic), (5) description of the specific kind of embodiment, (6) measurement items, and (7) embodiment dimensionality. Using this literature review, we could establish the entity (the whom/what) and the property (the relevant process/aspect) of the construct, which created the foundation for our construct definition [31,35,36,40]. The entity here is obviously a person. We described the property as experiencing wearable technology and perceiving an extension of oneself [4,17,41,42].

#### Dimensionality of the Construct and Construct Definition

After having defined the property and the entity of wearable technology embodiment, we explored the dimensionality of the concept [43-45]. Although most of the examined literature suggested the existence of multiple dimensions, there seemed to be no overall consensus on either the number or naming of the dimensions. We therefore decided to uncover the most plausible categorization by systematically collecting, juxtaposing, and comparing possible dimensions as mentioned in previous studies. This process, which is also known as structured conceptualization [44], resulted in the emergence of 3 clear themes: *body extension*, *cognitive extension, and self-extension*. These themes were not only the most popular topics but also covered the full spectrum of the individual (ie, body, mind, and sense of self). Body extension refers to a physical addition or replacement of the body. For example, a robotic hand that communicates touch to the end of the human limb, improving dexterity [46]. Cognitive extension, sometimes called cognitive prostheses [17,47], refers to the experienced extension of one’s cognitive capabilities such as navigation assistance or knowledge of the number of steps taken during a day. Finally, self-extension refers to an object being perceived as part of a person’s identity or sense of self. For example, experiencing a mobile phone as a representation or extension of yourself and personalizing the technology to be congruent with your self-image [11,15,48,49].

We then combined the entity (a human individual), property (experiencing wearable technology and perceiving an extension), and dimensionality of the construct (body extension, cognitive extension, and self-extension) to concisely define our construct [31]. This led to the following definition of *wearable technology embodiment*: A person experiencing technology worn on or near the body perceiving a certain extension of the body, cognition, or (sense of) self.

### Step 2: Development of Measures

#### Item Generation and Sorting

To come up with a first set of measurement items, we started the process of item generation, sorting, and selection (for a schematic overview of the item sorting and selection process, see Multimedia Appendix 1.) From our literature review, a first set of 24 preliminary items (12 body extensions, 7 cognitive extensions, 5 self-extensions; see Multimedia Appendix 2) was generated from the embodiment descriptions [43-45]. Some scales from the prosthesis literature were adapted to describe the extension of the body, which resulted in more items within this dimension. We then followed Hinkin and Tracey’s [50] content validity assessment approach to adapt and refine the wording of the 24 items. In total, 2 members of the research team independently assessed the items within each dimension and then compared their assessments to determine whether they tied in with our definition, sat in the correct dimension, and read clearly and concisely [31,51,52]. During this process, 6 items were removed (items 8 to 12 and 19, see Multimedia Appendix 2), which led to an updated item pool of 18 items.

#### Expert Interviews and Item Refinement

To judge the content validity of the 18 measurement items, we made use of 8 expert interviews [31,43,52,53] to examine each item for comprehension, applicability, and fit into the construct’s dimension [54]. In total, 2 of the experts worked as researchers at universities within human-computer interaction, one expert was an information systems researcher at a university and specialized in measurement scale development, 3 experts worked in wearable technology innovation and development, and 2 experts were individuals who were recently given wearables for the first time.

The experts were interviewed either in person or through a Skype conference call. After receiving a brief description and definition of wearable technology embodiment, they completed a Web-based survey, rating the applicability of each item from 1 (very inapplicable) to 5 (very applicable) and whether the item fits in the dimension. The experts chose whether they believed the item should be: (1) kept as is, (2) modified in a minor way, (3) modified in a major way, or (4) omitted [55]. Each item included an open field for suggestions or considerations [56]. The interviews ended with a brief discussion regarding the classification of 3 dimensions and possible suggestions for any new measurement items. The experts approved the dimensionality of our scale (ie, body extension, cognitive extension, and self-extension) and no new items were recommended. On the basis of their input, a few items were slightly reworded to improve their linguistic clarity. Using the feedback of the experts, 5 items were removed (items 5 to 7, 18, and 24; see Multimedia Appendix 2) because of repetition or lack of fit with the construct. This resulted in an updated measurement item pool of 13 items, which were subsequently used for empirical testing.

### Step 3: Method of Validation

To test the preliminary measurement instrument, data were collected through a vignette-based survey (see Multimedia Appendix 3). The sample consisted of a group of 182 undergraduate business students attending an e-Business course at a university in The Netherlands. Participation was voluntary and the students were offered a small incentive of 5 extra credit points on an exam worth 100 points by including their student number in the survey. Each respondent was given 3 different vignettes, describing the hypothetical situation using a different type of wearable technology with the purpose of tracking daily activities: a smart phone, a smart wristband, and a smart watch. The 3 devices were chosen because: (1) they are typical examples of wearable technology used in everyday life, being the most widely adopted technologies worn on or near the body, (2) they match our conceptualization and definition of *digital devices that incorporate wireless connectivity and allow the user to seamlessly access, interact with, and exchange information anywhere and anytime*, and (3) they fit into our delineation of user-centered preventive care wearable technology that can continuously and closely monitor the user’s motion and vital signs. Overall, the experience of participants with smart phones and relative unfamiliarity with activity tracking, smart wristbands, and smart watches supported our decision to make use of a vignette-based research design to confront them with the hypothetical use of wearable technology.

Each vignette asked the respondents to project themselves into the scenario of using the technology habitually to track their daily activities such as steps, hours of sleep, calorie burn, and achievement of personal health goals, regularly checking their progress throughout the day. The vignettes included pictures of the technology without brands to make the impression of the scenario as accessible as possible while avoiding branding bias. We randomized the order of the 3 vignettes for the respondents to avoid order bias. At the end of each vignette, the respondents were directed to a Web-based survey containing the 13 embodiment items (grouped into the 3 dimensions) and sociodemographic questions: age, gender, experience using the wearable technology (Table 2). To test the predictive validity of the construct [44], multi-item measurement instruments for trust, involvement, perceived usefulness, attitude toward use, and continuous intention also were included (see Multimedia Appendix 4 and full survey questions in attached documents). Before starting with a vignette, each respondent was told the length of time for the survey, who the investigator was, and the purpose of the study. The Web-based survey was pretested before sending it to the students, and included less than 8 questions per page, which were randomized to decrease order bias. Students were able to review or change their answers while filling in the survey. IP addresses were checked for multiple submissions and only completed questionnaires were analyzed. All surveys were checked for appropriate completion times.

**Table 2:** Sample characteristics (n=182).

Variables		Smartphone	Smart wristband	Smart watch
**Technology use,** **n (%)**				
	Own and use quite often to track activity	37 (20.1)	5 (2.7)	5 (2.7)
	Own but use seldom to track activity	72 (39.4)	7 (3.8)	6 (3.3)
	Own but do not use to track activity	72 (39.4)	3 (1.7)	3 (1.7)
	Do not own	2 (1.1)	168 (91.8)	169 (92.3)
**Age, n (%)**				
	18-20	—^a^	49 (26.9)	—
	21-23	—	105 (57.7)	—
	24-26	—	24 (13.2)	—
	27-30	—	4 (2.2)	—
**Gender, n (%)**				
	Female	—	68 (37.4)	—
	Male	—	114 (62.6)	—

^a^Same distribution.

## Results

### Step 4: Scale Evaluation and Refinement

#### Evaluate Goodness of Fit, Validity at Construct and Item Level, Eliminate Problematic Indicators

To test the dimensionality and further refine the scale items, we ran an exploratory factor analysis (EFA, principal component analysis with varimax rotation) on the set of 13 measurement items [57]. Aggregating the data of the 3 vignettes, each with 182 respondents, led to a sample that contained 546 responses. In total, 3 items were removed from the analysis (items 4, 16, and 17; see Multimedia Appendix 2) as they loaded substantially on 2 dimensions or more [58]. Rerunning the EFA with the 10 remaining items confirmed the 3 dimensions of wearable technology embodiment (Kaiser-Meyer-Olkin Measure of sampling adequacy ((0.80) Bartlett’s test of sphericity 2390, P<.001) and accounted for 71.22% of the variance. All items loaded significantly on only 1 dimension and all factor loadings were above the recommended threshold value of 0.50 [58]; therefore, providing first evidence of the convergent and discriminant validity of the measurement instrument. As the largest factor within the EFA explained less than 50% of the variance (28.5%), evidence for common method bias was not found [31,59].

To further test the measurement instrument, we performed a confirmatory factor analysis (CFA) [60,61] using the software package IBM, SPSS Amos 23 [57] (maximum likelihood estimation). We tested the EFA solution of 3 dimensions as intercorrelated first-order factors [61]. See Table 3 for: Chi square degrees of freedom calculated probability, Minimum Discrepancy Degrees of Freedom (CMIN/ *df*), Goodness of Fit (GFI), Adjusted Goodness of Fit (AGFI)), Normed Fit Index, Incremental Fit Index, Tucker Lewis Index (TLI), Comparative Fit Index (CFI), root mean square error of approximation (RMSEA), Akaike information criterion, Browne- Cudeck Criterion (BCC), Bayesian Information Criterion (BIC). After removing one item (item 23, see Multimedia Appendix 2) to improve the model fit, the found 9-item solution demonstrated a good fit with the data (CMIN*/df*) <5; GFI, AGFI, Normed Fit Index Incremental Fit Index (NFI), TLI, CFI >0.90; RMSEA <0.08; Table 3).

**Table 3:** Confirmatory factor analysis alternative model testing.

Model	Chi square (*df*)	*P* value	CMIN/df^a^	GFI^b^	AGFI^c^	NFI^d^	IFI^e^	TLI^f^	CFI^g^	RMSEA^h^	Akaike information criterion	BCC^i^	BIC^j^
3 first-order correlated	104.26 (24)	<.001	4.345	.96	.93	.94	.96	.94	.96	.078	146.26	147.05	236.62
3 first-order uncorrelated	273.79 (27)	<.001	10.141	.91	.85	.86	.87	.83	.87	.130	309.79	310.46	387.24
One first-order factor	812.73 (27)	<.001	30.101	.72	.53	.60	.61	.48	.61	.231	848.73	849.41	926.18

^a^CMIN/df: Minimum Discrepancy Degrees of Freedom.

^b^GFI: Goodness of Fit.

^c^AGFI: Adjusted Goodness of Fit Index.

^d^NFI: Normed Fit Index.

^e^IFI: Incremental Fit Index.

^f^Tucker Lewis Index.

^g^CFI: Comparative Fit Index.

^h^RMSEA: root mean square error of approximation.

^i^BCC: Browne-Cudeck Criterion.

^j^BIC: Bayesian Information Criterion.

### Step 5: Assess Scale Validity

#### Test Alternative Models, Assess Convergent and Discriminant Validity

To further test the applicability of our dimensions, we tested 2 alternative models [52]: a model of 3 uncorrelated first-order factors and a model treating the 9 items as indicators of 1 first-order factor. The CFA results of the alternative models clearly showed that the alternative models did not have a good fit with the data (CMIN/*df*>5; GFI, AGFI, NFI, TLI, CFI<0.90; RMSEA<0.08; Akaike information criterion, BCC, BIC>scores of 3 first-order correlated model). These outcomes confirm that wearable technology embodiment is best modeled as a set of 3 correlated, first-order factors. In addition, the very poor fit of the 1 first-order factor reconfirmed the absence of common method bias.

The convergent validity of the 3 dimensions and their reliability (Table 4) was confirmed via the CFA factor loadings (>0.70) [58], average variance extracted (AVE) values (>0.50) [53], and minimum item to total correlations (>0.40) [62], which exceeded the established threshold value. The reliability of the dimensions was also confirmed as Cronbach alpha and composite reliability exceeded 0.70. The discriminant validity (Table 5).could be confirmed as the AVE of each construct exceeded the values of the crossconstruct squared correlations [63,64] .

**Table 4:** Convergent validity: Factor loadings, Cronbach alphas, composite reliabilities, (average variance extracted), and minimum item to total correlation.

Dimension and item		Factor loading (CFA)	Cronbach alpha	Composite reliability	Average variance extracted	Minimum item to total correlation
**Body extension**		—^a^	0.84	0.88	0.71	0.76
	When using a <technology> it feels like it is part of my body	0.83	—	—	—	—
	When using a <technology> it feels like it is an extension of my body	0.74	—	—	—	—
	When using a <technology> it almost feels like it is incorporated into the body	0.86	—	—	—	—
**Cognitive extension**		—	0.72	0.84	0.64	0.80
	Using <technology> heightens my knowledge about my activity	0.61	—	—	—	—
	Using <technology> helps me learn about my activity	0.84	—	—	—	—
	Using <technology> helps me gain understanding of my activity	0.62	—	—	—	—
**Self-extension**		—	0.86	0.88	0.71	0.76
	When using a <technology> it feels like it is an extension of myself	0.76	—	—	—	—
	When using a <technology> it feels like it is related to my sense of self	0.86	—	—	—	—
	When using a <technology> it feels like it is a psychological extension of myself	0.81	—	—	—	—

^a^Not applicable.

**Table 5:** Discriminant validity testing: average variance extracted (italics) versus crossconstruct squared correlations between the constructs.

Constructs	Body extension	Cognitive extension	Self-extension	Trust	Involvement	Perceived usefulness	Attitude toward use	Continuous intention
Body extension	*0.87* ^a^	—^b^	—	—	—	—	—	—
Cognitive extension	0.13	*0.80*	—	—	—	—	—	—
Self-extension	0.53	0.12	*0.88*	—	—	—	—	—
Trust	0.24	0.50	0.14	*0.86*	—	—	—	—
Involvement	0.13	0.11	0.33	0.01	*0.91*	—	—	—
Perceived usefulness	0.17	0.60	0.15	0.61	0.13	*0.86*	—	—
Attitude toward use	0.27	0.41	0.25	0.53	0.29	0.60	*0.84*	—
Continuous intention	0.18	0.24	0.25	0.35	0.49	0.40	0.59	*0.90*

^a^Italic scores (diagonal) are the average variance extracted of the individual constructs.

^b^Not applicable.

#### Test of Predictive Validity

To test the predictive validity of the wearable technology embodiment instrument, we utilized 5 dependent variables related to technology adoption: trust [55], involvement [65], perceived usefulness [66], attitude toward use [67], and continuous intention [68]. Structural equation modeling was used to test the extent to which the 3-dimensional wearable technology embodiment construct explained each of the 5 dependent variables by making use of the software package IBM SPSS Amos 23 [57] (maximum likelihood estimation).

The results of the analyses (see Multimedia Appendix 5) confirm a good fit with the data as the GFI, AGFI, NFI, TLI, and CFI exceed the recommended value of 0.90 and the RMSEA does not surpass the value of 0.08 [58]. The 3 dimensions of wearable technology embodiment explain considerable (trust and perceived usefulness) to acceptable amounts of the variance (involvement, attitude, and continuous intention) of the dependent variables [69]. Except for the influence of body extension on the attitude (beta=.17: *P*=.004), the standardized paths imply that the found influences account for a substantial proportion of the variance [70] (beta>.20). Overall, the results confirm the predictive validity of both the multidimensional construct and individual dimensions of the wearable technology embodiment instrument.

## Discussion

Utilizing the development and validation process as prescribed by Mackenzie et al [31], we made use of literature study, expert interviews, and empirical data collected for 3 wearable technologies to conceptualize *wearable technology embodiment* and build a measurement instrument. We established wearable technology embodiment as a 3-dimensional concept consisting of the dimensions: body extension, cognitive extension, and self-extension.

### Academic Implications

The findings of this study have 2 implications for academic research. First, the development of a measurement instrument serves researchers by quantifying the perception of wearable technology extending the user’s body, cognitive capacity, and sense of self. Researchers could develop a mixed methods approach to extend qualitative findings or compare usage data in upcoming studies. Second, the results of the predictive validity testing suggest that wearable technology embodiment significantly adds to well-known constructs that have been applied previously to study the adoption and use of new technology (ie, trust, involvement, perceived usefulness, attitude toward use, and continuous intention). By adding to these constructs, wearable technology embodiment seems to function as a valuable extension of theoretical structures such as the technology acceptance model [71], theory of reasoned action [72], and expectation confirmation theory [73].

### Practical Implications

Our results also have practical implications. Given that the dimensions of wearable technology embodiment seem to contribute positively to perceptions of trust, involvement, usefulness, and behavioral attitudes and intentions, wearable technology developers could benefit from this knowledge by developing devices in such as a way that they better fit the user’s body shape (body extension), improve the acceptability of biofeedback data [74] (cognitive extension), and heighten customizable features and fashion [75-77], connecting to the user’s personal identity (self-extension). Users are likely to evaluate the wearable technology more positively with customizations focused on body, cognition, and self-extension. Furthermore, wearable technology developers could make use of the outcomes of the predictive validity at the dimension level to further prioritize their efforts. For example, if the objective is to generate more trust in the technology, it seems advisable to focus on cognitive extension(s) as this was the strongest trust determinant in our model. When the aim is to generate more involvement, however, a focus on designing and developing self-extension(s) seems a better choice. Overall, integrating the 3 wearable technology embodiment dimensions into design and development priorities can aid practitioners in making more effective decisions.

### Limitations and Recommendations

This study has been subject to a couple of limitations that could guide scholars in setting up future research. First, the sample consisted of a rather homogeneous group of students that can be classified as millennials, most likely raised with technology [78], who are active users of emerging technologies [79]. The selection and use of this sample seems justified given that it reduces the likelihood that differences between the respondents such as age, educational background, and technological savviness may have biased our findings [80,81]. Furthermore, it is in line with the key objective of our work to setup and test a theoretically meaningful construct instead of generalizing found research effects to larger populations [82]. This is not to say, however, that research would not benefit from using the developed measurement instrument in future effect application studies with different, more heterogeneous samples [80]. We foresee this research as a next step in the field of technology embodiment studies.

Second, by making use of a vignette method, we were able to confront the respondents with the situation of using different forms of wearable technology. The use of the vignette method has several advantages. It simulates realism, can be tailored to the specific research problem [83], does not require respondents to have in-depth knowledge of the presented stimuli, and reduces the likelihood of confounding effects since participants respond to the same stimulus [84]. Still, it cannot reflect all facets of actual usage situations and we, therefore, suggest researchers to apply and crossvalidate the instrument in a real-word context.

Third, this study was framed within the context of using wearable technology to track daily activities such as movement, hours of sleep, calorie burn, and personal health goals. Past studies have highlighted that low adherence to mobile interventions is a common occurrence [85,86], yet the opportunity to measure and address health concerns is evident [64]. Our findings regarding embodiment positively influence constructs related to technology adoption (ie, trust, involvement, usefulness, attitude, and continuous intention) and suggest that this embodiment scale could give insight into a determination as to which individuals will adhere to the intervention. Predicting adherence levels and identifying individuals unlikely to adhere could help in understanding and possibly improving the low adherence rates during mobile and wearable health interventions.

Fourth, even though we do find that wearable technology embodiment functions as a determinant of constructs rooted in different theoretical frameworks, the focus of this study was on conceptualization and measurement and not on the extension of nomological networks. More theoretical rationale and validation are needed to substantiate our findings. We invite researchers to adopt the concept of wearable technology embodiment in their future studies.

Fifth, this study focused on some of the most popular wearables in consumer technology, which are used for preventative health care (ie, smart phone, activity tracker, and smart watch). Other devices that serve this purpose include smart textiles, tattoos, and jewelry. Next to these kind of wearables, the developed measurement instrument also could be applied to user-centered disease monitoring wearable devices such as wearable cameras that enhance chronic disease self-management [87], insulin monitors and pumps in the treatment of diabetes [19], smart gloves that assist rheumatoid arthritis patients in applying therapy [88], and medical-grade electrocardiogram wristwatches that assist cardiac patients to detect heart arrhythmia [89]. Furthermore, although our inquiry did not focus on care provider centered wearables, it seems logical that the measurement instrument could apply to wearable aids that are used during medical procedures such as Google glasses in surgery [28]. Still, more empirical exploration is needed to validate the applicability of our instrument for these kinds of wearable technology. We encourage researchers to do so in future studies.

## Appendix

Multimedia Appendix 1 Flow diagram measurement item selection.

Multimedia Appendix 2 Measurement items in stages of development.

Multimedia Appendix 3 Survey vignettes-smartphone, smart wristband, and smart watch.

Multimedia Appendix 4 Constricts and items predictive validity testing.

Multimedia Appendix 5 Results predictive validity testing.

## Abbreviations

AGFI: Adjusted Goodness of Fit
AVE: average variance extracted
BCC: Browne-Cudeck Criterion
BIC: Bayesian Information Criterion
CFA: confirmatory factor analysis
CFI: Comparative Fit Index
CMIN/df: Minimum Discrepancy Degrees of Freedom
EFA: exploratory factor analysis
GFI: Goodness of Fit
mHealth: mobile health
NFI: Normed Fit Index Incremental Fit Index
RMSEA: root mean square error of approximation
TLI: Tucker Lewis Index
