# Supplementary figures and images for: Is Wearable Technology Becoming Part of Us? Developing and Validating a Measurement Scale for Wearable Technology Embodiment
Source: JMIR Mhealth Uhealth. 2019 Aug 9;7(8):e12771. doi: 10.2196/12771 (PMC6709898; doi:10.2196/12771)

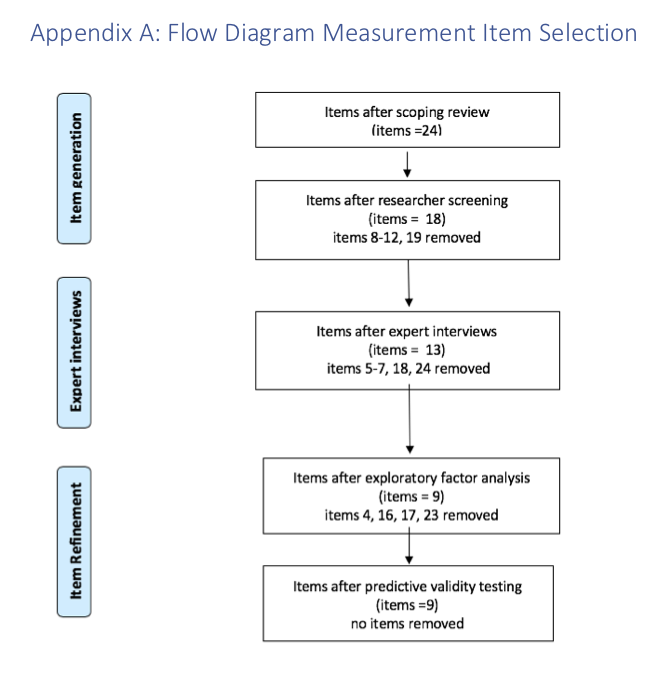

Supplement: Multimedia Appendix 1 [file mhealth_v7i8e12771_app1.png]

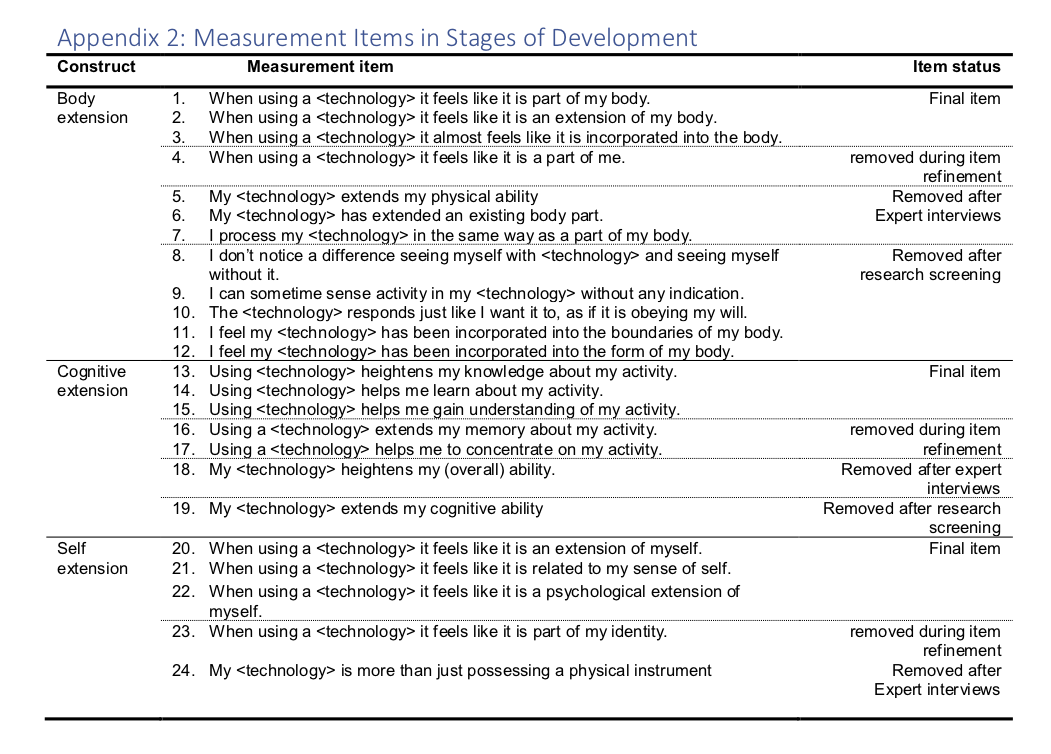

Supplement: Multimedia Appendix 2 [file mhealth_v7i8e12771_app2.png]

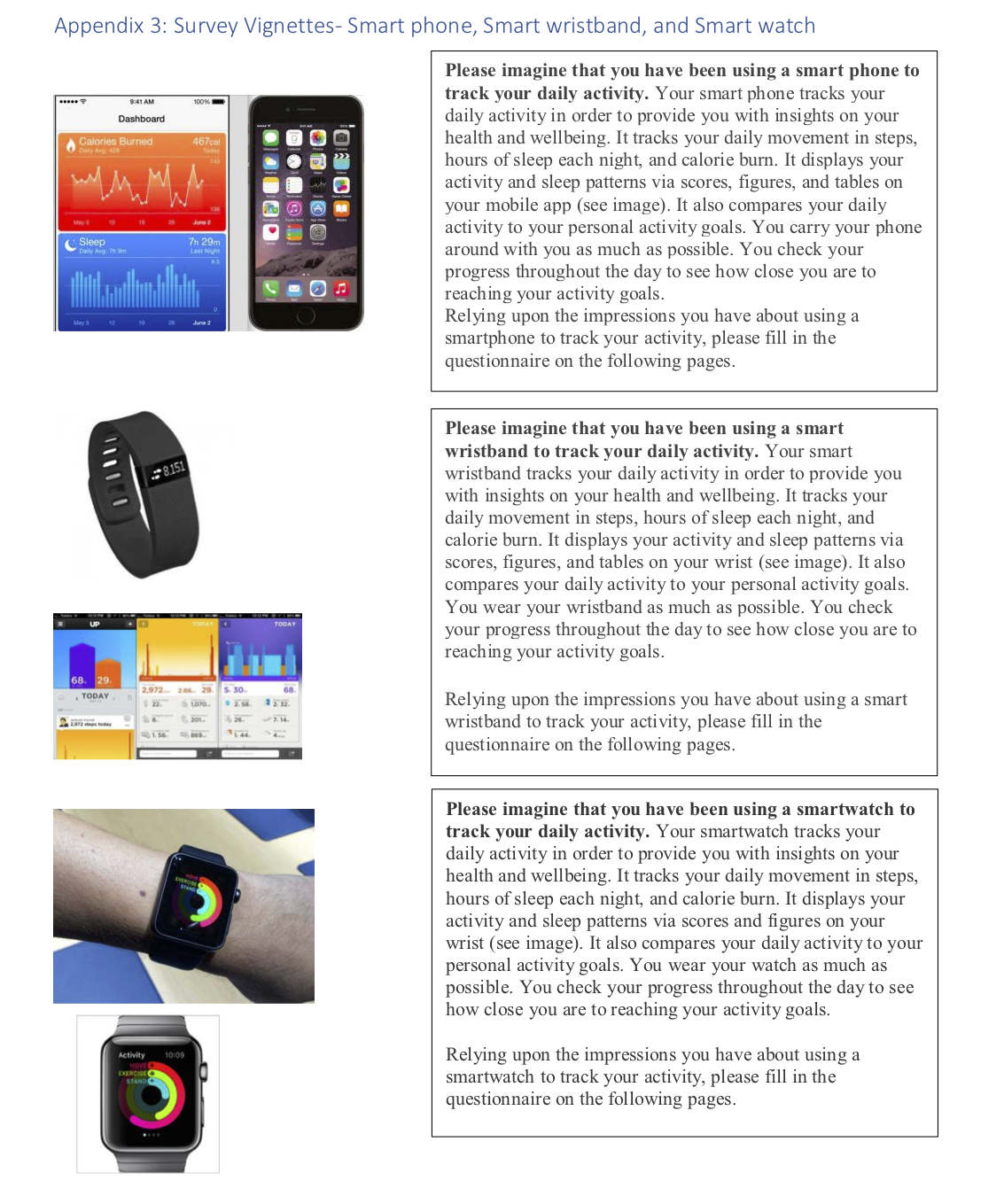

Supplement: Multimedia Appendix 3 [file mhealth_v7i8e12771_app3.png]

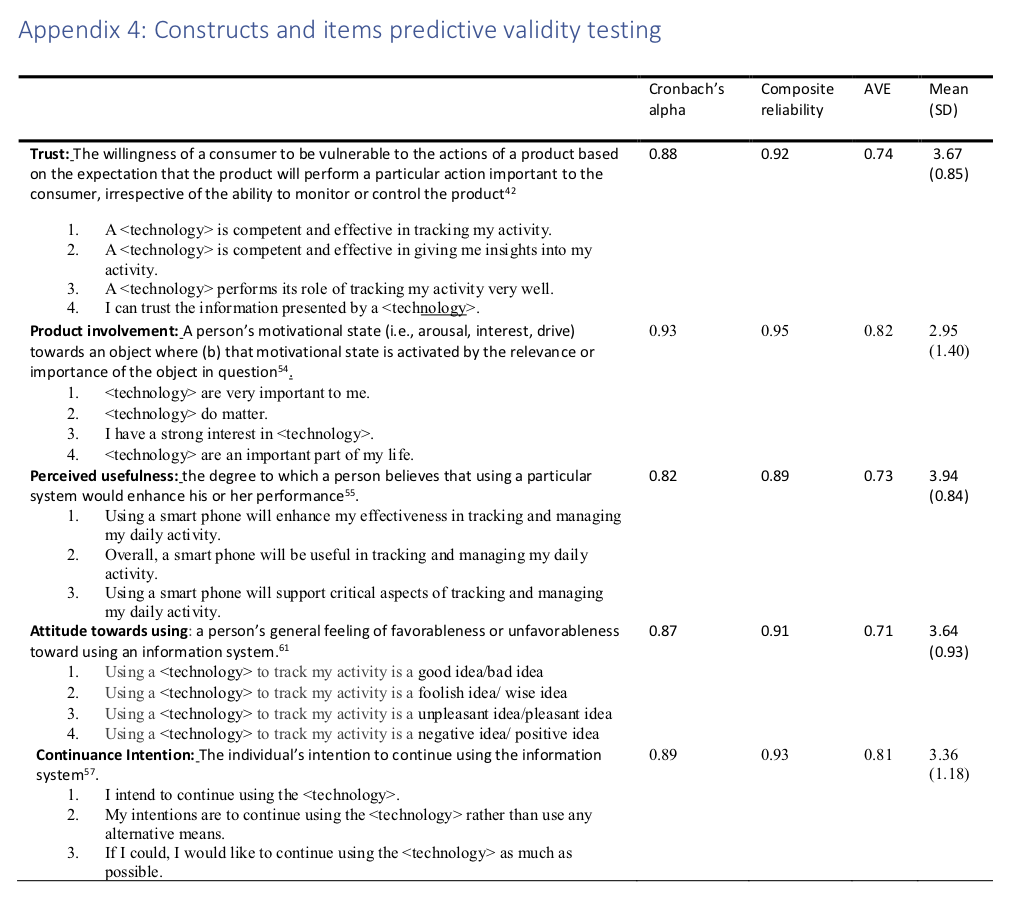

Supplement: Multimedia Appendix 4 [file mhealth_v7i8e12771_app4.png]

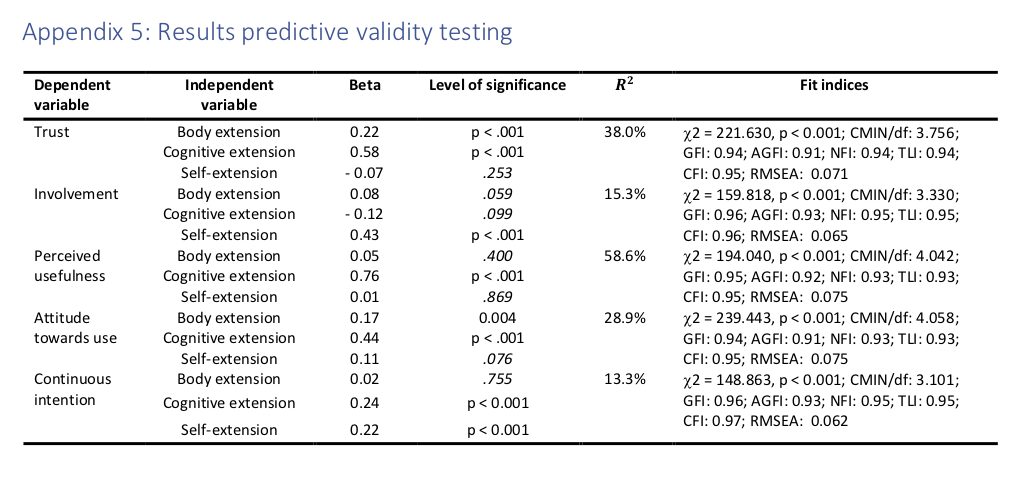

Supplement: Multimedia Appendix 5 [file mhealth_v7i8e12771_app5.png]
